# Supplementary material for: The nucleolus is the site for inflammatory RNA decay during infection
Source: Nat Commun. 2022 Sep 3;13:5203. doi: 10.1038/s41467-022-32856-2 (PMC9440930; doi:10.1038/s41467-022-32856-2)
Supplement: Supplementary file 1 — Supplementary Information [file 41467_2022_32856_MOESM1_ESM.pdf]

The nucleolus is the site for inflammatory RNA decay during infection

Taeyun A. Lee[1], Heonjong Han[1,2], Ahsan Polash[3], Seok Keun Cho[1], Ji Won Lee[4], Eun A. Ra[1], Eunhye Lee[1], Areum Park[1], Sujin Kang[1], Junhee L. Choi[1], Ji Hyun Kim[5], Ji Eun Lee[5,6], Kyung-Won Min[4,7], Seong Wook Yang[1], Markus Hafner[3], Insuk Lee[2], Je-Hyun Yoon[7], Sungwook Lee[8], Boyoun Park[1]

Supplementary Information

Supplementary Figures

Supplementary Figure legends

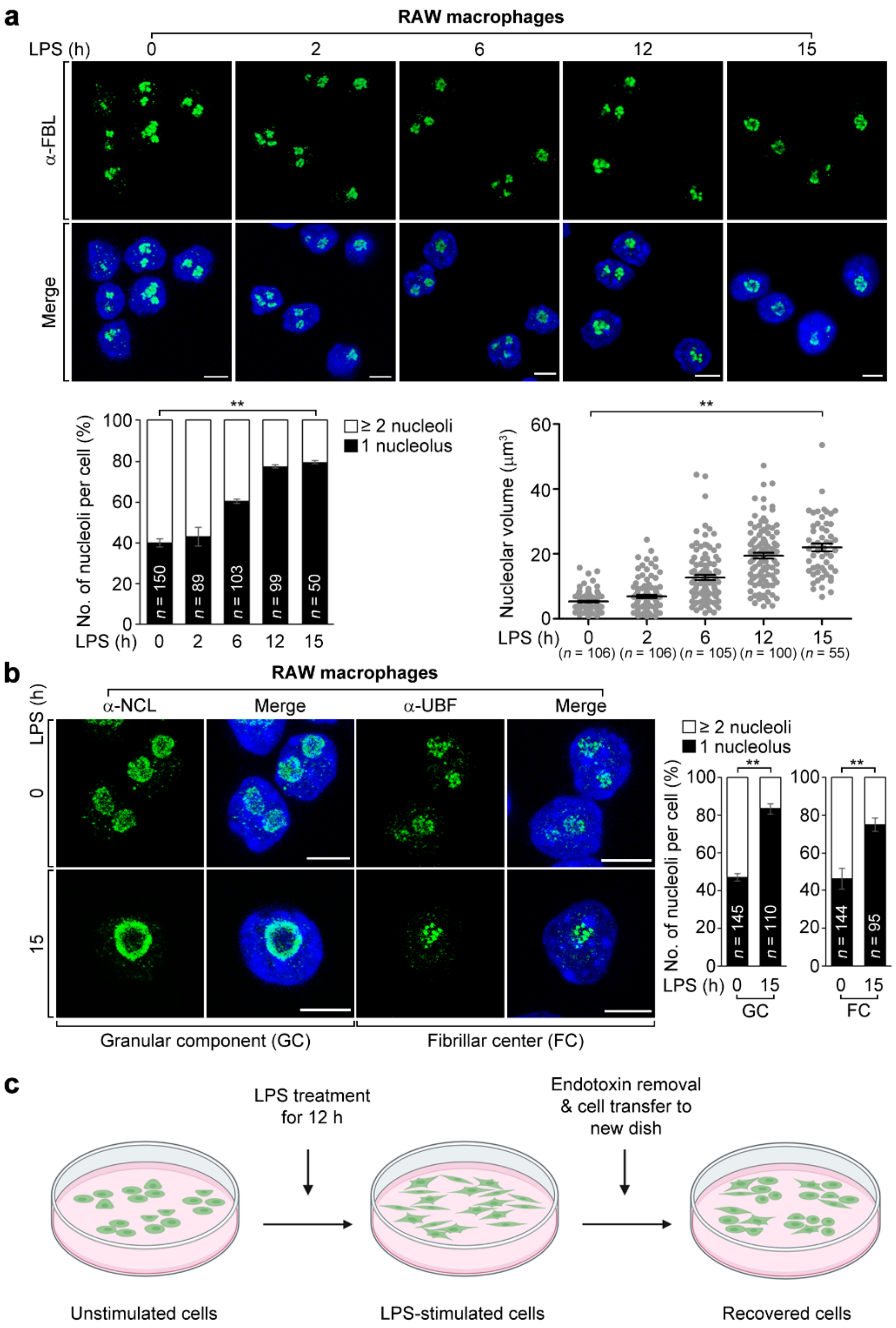

Supplementary Figure 1 | LPS induces nucleolar fusion. a, **b**, Images showing nucleolar fusion at the indicated times of LPS (80 ng ml^{-1}) stimulation in RAW 264.7 macrophages. NCL, FBL, and UBF indicate three nucleolar compartments, the granular component (GC), dense fibrillar component (DFC), and fibrillar center (FC), respectively. Graphs representing the percentage of cells with indicated nucleoli numbers per cell or the volume of nucleoli. *n*, total cells counted. Blue, DAPI. Scale bars, 5 μm. *P* values are determined by unpaired two-tailed *t*-test. $^{**}P<0.01$ (Student's *t*-test). **c**, Schematic outline of experimental design for recovery from LPS stimulation. LPS-stimulated RAW 264.7 cells were transferred to a new plate with fresh media after endotoxin washout. After incubation for 24 h, the recovered cells were subjected to IF assay to observe their nucleolar morphology. Schematic diagrams were created with BioRender.com. All data are representative of three independent experiments and bar graphs (**a** and **b**) or dot plot (**a**) are presented as means ± s.d. or s.e.m., respectively. Source data are provided as a Source Data file.

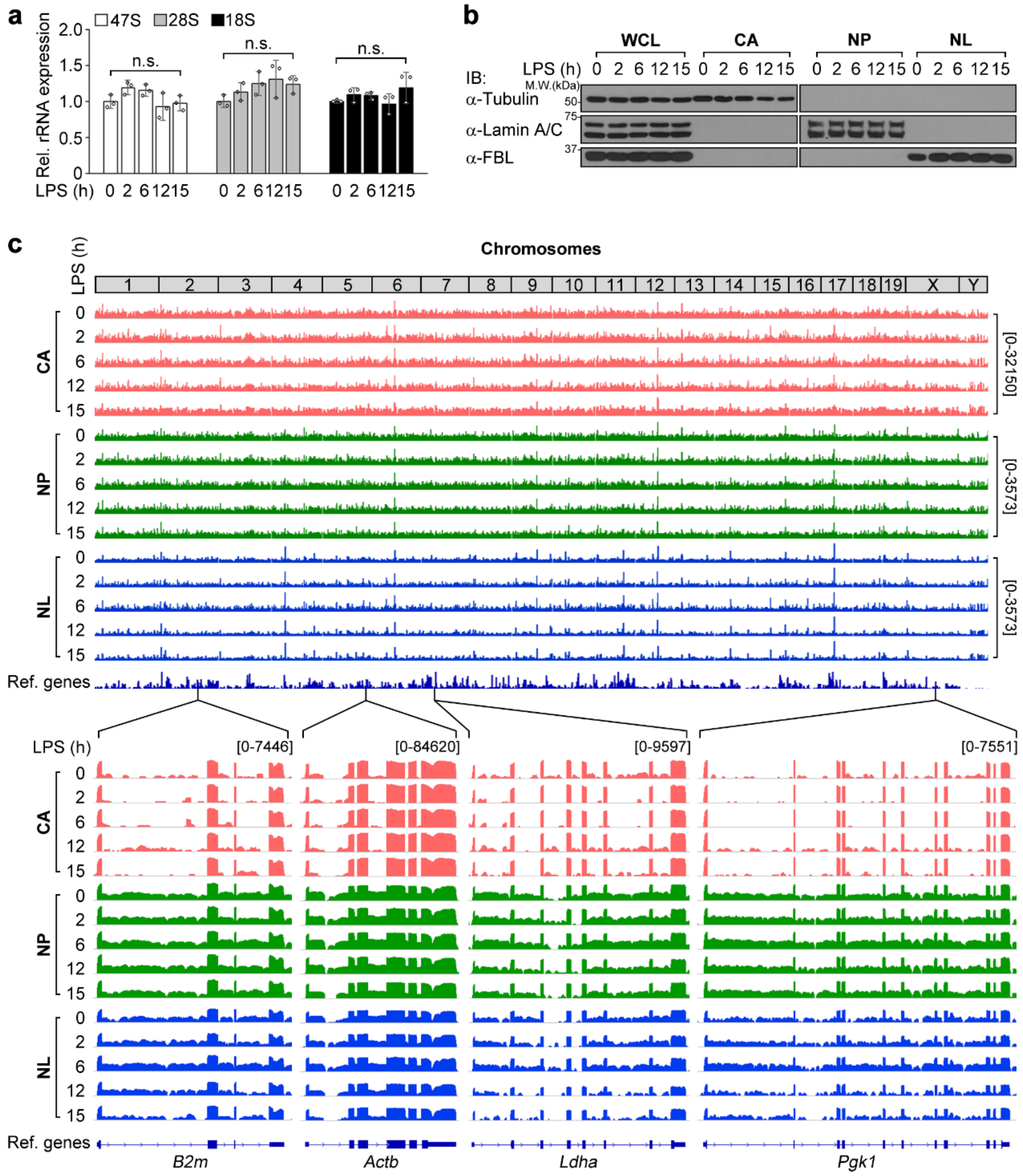

Supplementary Figure 2 | Nucleoli contain a large number of introns and exons of protein-coding genes. a, Graph showing 47S, 28S, and 18S rRNA levels in macrophages following the time course of LPS stimulation. *Gapdh* mRNA was used for normalization. *P* values are determined by unpaired two-tailed *t*-test with multiple comparisons. n.s., not significant. **b**, High-purity of cytoplasmic (CA), nucleoplasmic (NP), or nucleolar (NL) fractions from macrophages stimulated by LPS during the indicated times is analyzed by immunoblots with antibodies recognizing specific marker proteins, tubulin (CA), lamin A/C (NP), or FBL (NL). These fractions were used for RNA-seq results in Figs. 2a-d and Supplementary Fig. 2c. WCL, whole cell lysates. **c**, IGV displaying the read alignment to mouse reference genes in each fraction and the intronic and exonic read density of housekeeping genes (*B2m*, *Actb*, *Ldha*, and *Pgk1*). Coverage tracks in red, green, and blue indicate intronic or exonic read density in the CA, NP, and NL fractions, respectively. Data are representative of three independent experiments and are presented as mean ± s.d. in **a**. Source data are provided as a Source Data file.

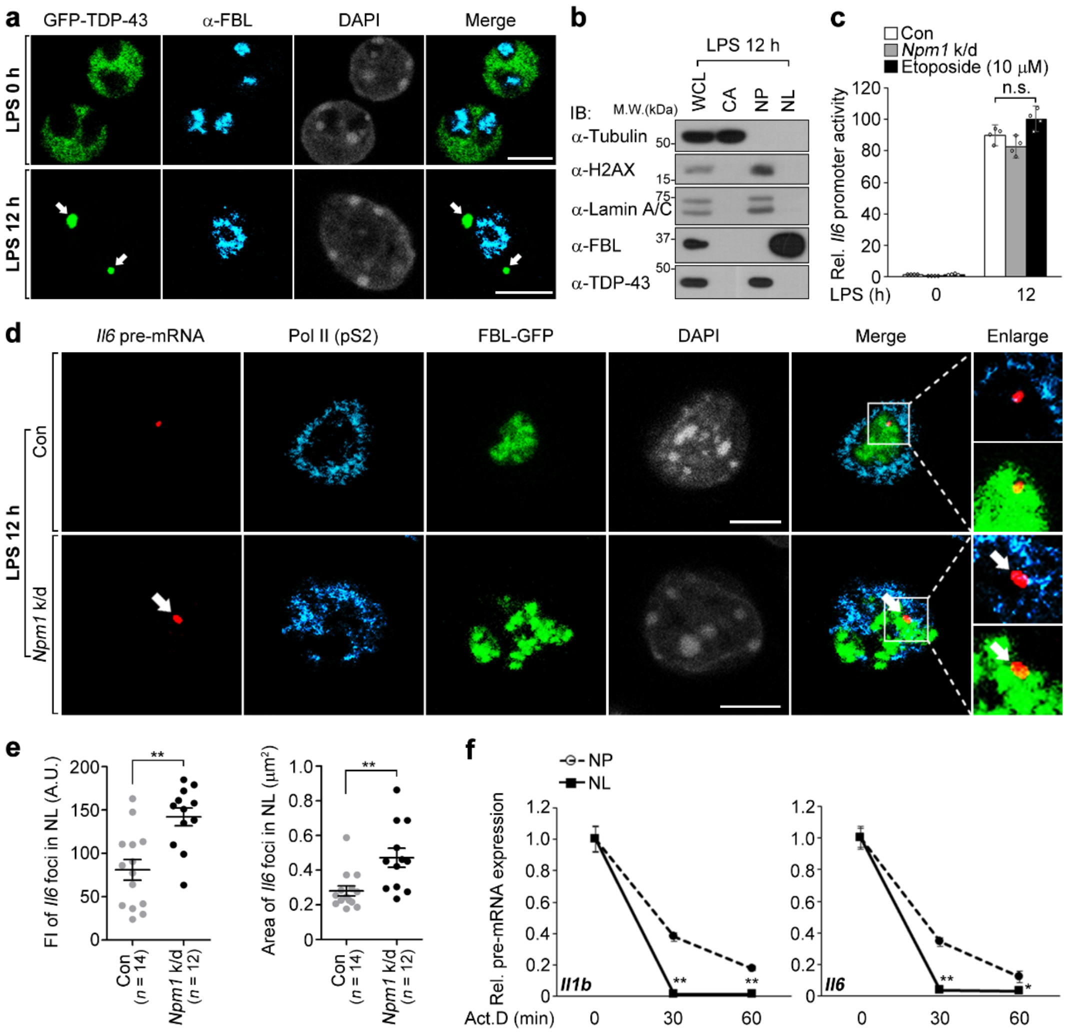

Supplementary Figure 3 | The nucleolus is related to modulate inflammatory pre-mRNA stability rather than transcription or splicing. a, Images showing that InSAC is separated from the nucleolus in GFP-TDP-43-expressing RAW 264.7 cells in response to 12 h LPS. White arrows indicate the InSAC. DAPI, gray. Scale bars, 5 μm. **b**, Immunoblots showing the spatial preference of InSAC that is detectable in the nucleoplasmic fraction, but not in nucleolar fraction in 12 h LPS-stimulated RAW 264.7 cells. This is analyzed with antibodies recognizing specific marker proteins, tubulin (CA), H2AX (NP), lamin A/C (NP), or FBL (NL). **c**, Luciferase assay showing the *Il6* promoter activity in *Npm1*-depleted or etoposide-treated RAW 264.7 macrophages with or without 12 h LPS stimulation. n.s., not significant. **d**, RNA-FISH showing the effect of *Npm1* depletion on the spatial preference of *Il6* pre-mRNAs between the nucleolus and active transcription sites in *Npm1*-depleted RAW 264.7 cells expressing FBL-GFP under 12 h LPS stimulation. White arrows indicate that inflammatory *Il6* pre-mRNAs foci were overlapped with the nucleolus. White boxes delineate the area of enlargement showing localization of *Il6* pre-mRNA foci. DAPI, gray. Scale bars, 5 μm. **e**, Graphs showing the fluorescence intensity and area of *Il6* pre-mRNA foci in the nucleolus. *n*, total foci counted. **f**, Graphs showing the half-life of *Il1b* and *Il6* inflammatory pre-mRNAs in the nucleoplasmic or nucleolar fractions from 12 h LPS-stimulated RAW 264.7 cells after Act.D treatment by performing RT-qPCR using intron-exon primer pairs. *P* values are determined by unpaired two-tailed *t*-test. *P<0.05; **P<0.01 (Student's *t*-test). Data are representative of three independent experiments and are presented as mean ± s.d. (**c** and **f**) or ± s.e.m. (**e**). Source data are provided as a Source Data file.

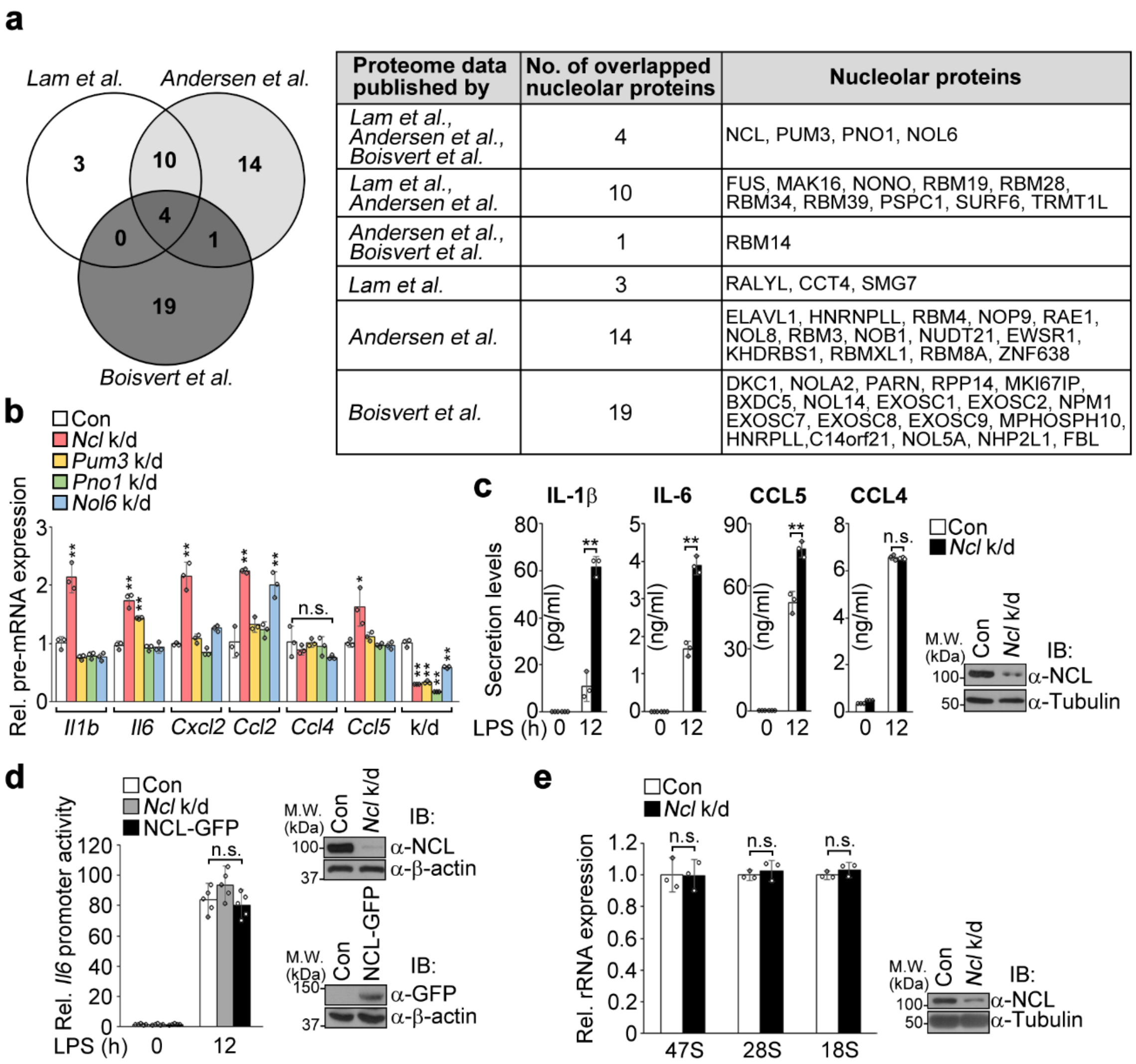

Proteome data published by	No. of overlapped nucleolar proteins	Nucleolar proteins
Lam et al., *Andersen et al.*, *Boisvert et al.*	4	NCL, PUM3, PNO1, NOL6
Lam et al., *Andersen et al.*	10	FUS, MAK16, NONO, RBM19, RBM28, RBM34, RBM39, PSPC1, SURF6, TRMT1L
Andersen et al., *Boisvert et al.*	1	RBM14
Lam et al.	3	RALYL, CCT4, SMG7
Andersen et al.	14	ELAVL1, HNRNPLL, RBM4, NOP9, RAE1, NOL8, RBM3, NOB1, NUDT21, EWSR1, KHDRBS1, RBMXL1, RBM8A, ZNF638
Boisvert et al.	19	DKC1, NOLA2, PARN, RPP14, MKI67IP, BXDC5, NOL14, EXOSC1, EXOSC2, NPM1 EXOSC7, EXOSC8, EXOSC9, MPHOSPH10, HNRPLL,C14orf21, NOL5A, NHP2L1, FBL

Supplementary Figure 4 | NCL levels are related to inflammatory gene expression. a, Venn diagram visualizing the overlapping nucleolar proteins capable of both binding RNAs and translocating to various subcellular locations between three public nucleolar proteome datasets (*Lam et al.*, *Andersen et al.*, and *Boisvert et al.*). **b**, Graphs showing inflammatory RNA levels and knockdown efficiency of target genes in *Ncl*-, *Pum3*-, *Pno1*-, or *Nol6*-depleted RAW 264.7 macrophages under 12 h LPS stimulation. **c**, Effect of *Ncl* depletion on levels of the secreted inflammatory proteins in 12 h LPS-stimulated RAW 264.7 macrophages. **d**, Influence of *Ncl* levels on *Il6* promoter activity measured by luciferase assay of *Ncl*-depleted or NCL-GFP-overexpressing RAW 264.7 cells under 12 h LPS stimulation. **e**, Effect of *Ncl* depletion on levels of rRNAs in 12 h LPS-stimulated RAW 264.7 cells. All experiments of *Ncl* depletion were performed upon 2 days of retroviral transduction. *Gapdh* mRNA was used for normalization of rRNA levels. Immunoblots showing the protein expression level of endogenous or GFP-tagged NCL in RAW 264.7 cells (**c-e**). *P* values are determined by unpaired two-tailed *t*-test. **P*<0.05, ***P*<0.01 (Student's *t*-test). n.s., not significant. Data are representative of three independent experiments and are presented as mean ± s.d. in **b-e**. Source data are provided as a Source Data file.

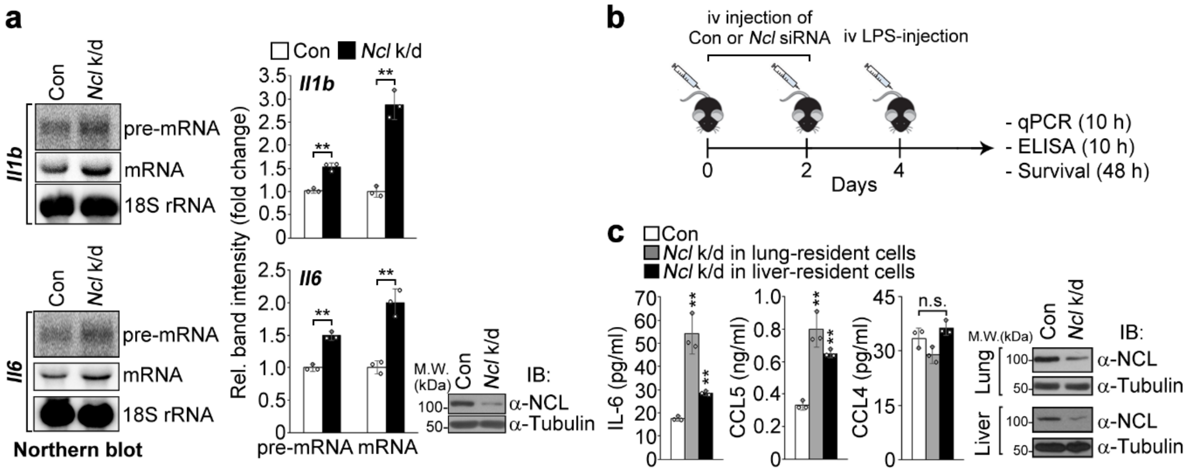

Supplementary Figure 5 | NCL promotes inflammatory RNA instability. **a**, Northern blot analysis showing both pre-mRNA and mRNA levels of *Il1b* or *Il6* in *Ncl*-depleted RAW 264.7 macrophages under 12 h LPS stimulation. Right graph plotting quantification of northern blot data. **b**, Schematic outline of experimental design for *in vivo* experiments. For details on *in vivo* experiment, see Methods. iv, intravenous. **c**, Graphs or immunoblots showing protein secretion levels of inflammatory genes or *Ncl* knockdown efficiency in lung- or live-resident cells of wild-type or *Ncl*-depleted mice after LPS challenge. *P* values are determined by unpaired two-tailed *t*-test. ***P*<0.01 (Student's *t*-test). n.s., not significant. Data are representative of three independent experiments and are presented as means ± s.d. in **a** and **c**. Source data are provided as a Source Data file.

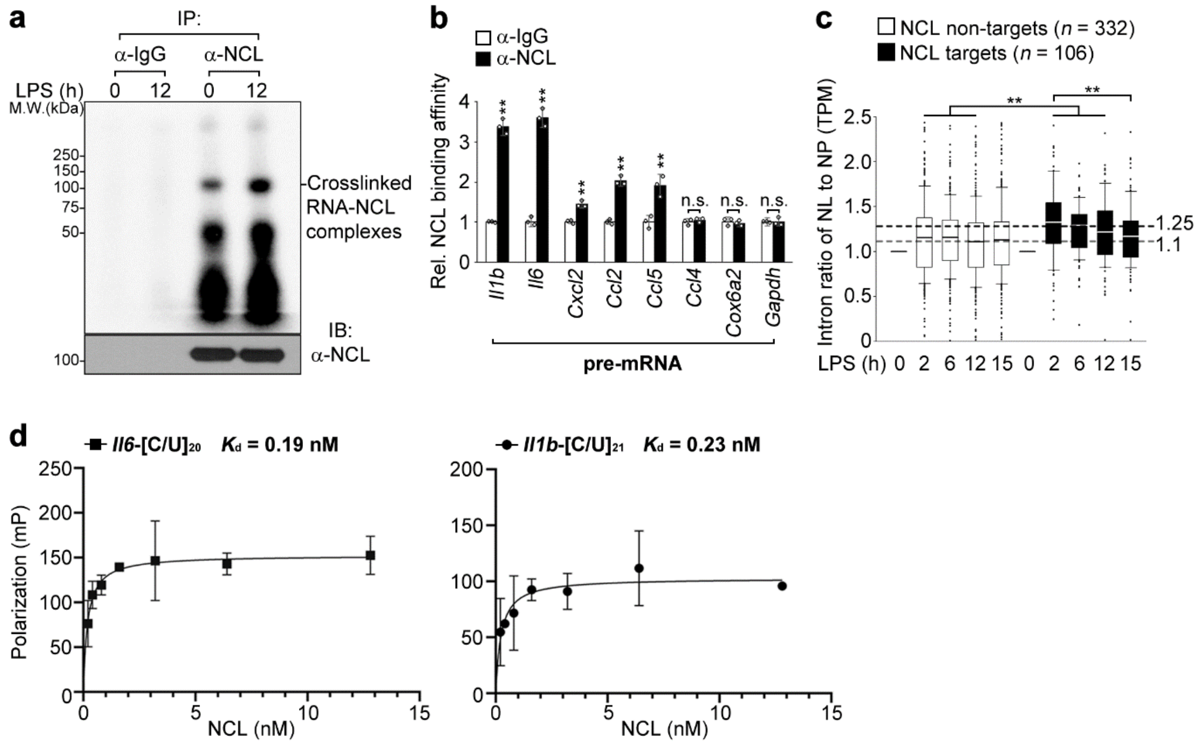

Supplementary Figure 6 | NCL directly binds the C/U-rich sequence in the intronic regions of inflammatory RNAs. a, Phosphorimage showing crosslinked RNA-NCL complexes in unstimulated or LPS-stimulated RAW 264.7 macrophages. For details on PAR-CLIP experiment, see Methods. **b**, RIP-qPCR showing the interaction of NCL with inflammatory pre-mRNAs, but not *Ccl4*, in 12 h LPS-stimulated RAW 264.7 cells. *Gapdh* pre-mRNA was used for normalization of inflammatory and non-inflammatory pre-mRNAs. *P* values are determined by unpaired two-tailed *t*-test. $^{**}P<0.01$ (Student's *t*-test). n.s., not significant. **c**, Time-course changes in nucleolar enrichment of intronic reads between NCL-targeted (n = 106) and non-targeted (n = 332) genes based on presence or absence of C/U-rich sequences in their intronic regions. Dotted lines and numbers represent average TPM values from samples of 2, 6, 12, and 15 h of LPS stimulation. The box plot represented 5th, 25th, 50th, 75th and 95th percentiles, with median values labeled. $^{**}P<0.01$ (Two-tailed Mann-Whitney *U* test). **d**, Anisotropy analysis graphs represent the binding affinity of GST-NCL to *Il1b* or *Il6* RNA probes and are presented as means ± s.d. of two independent experiments. PAR-CLIP experiment is representative of three independent experiments and are presented as means ± s.d. in **b**. Source data are provided as a Source Data file.

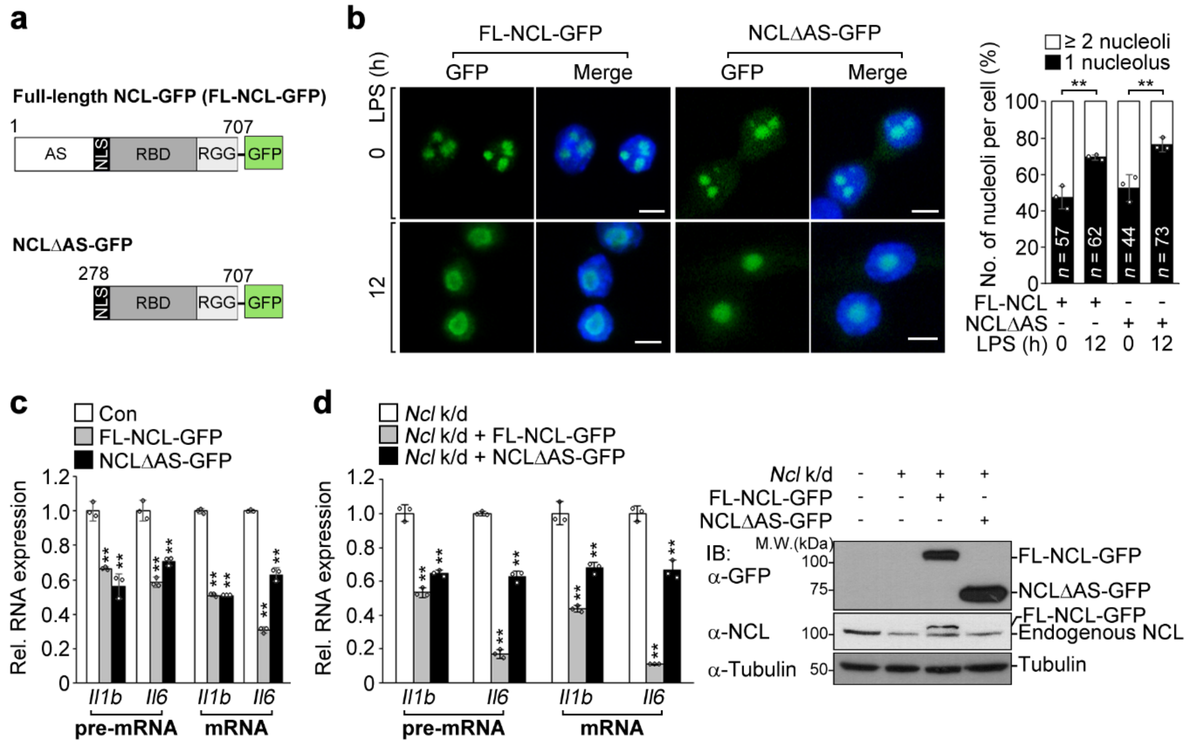

Supplementary Figure 7 | The N-terminal domain of NCL containing acidic stretches is dispensable for its activity on LPS-mediated nucleolar fusion or inflammatory RNA instability. **a**, Domain architecture of mouse NCL. AS, acidic stretches; NLS, nuclear localization signal; RBD, RNA-binding domain; RGG, arginine/glycine-rich region. **b**, **c**, Effects of GFP-tagged wild-type (FL-NCL-GFP) or mutant NCL lacking acidic regions (NCLΔAS-GFP) on nucleolar fusion (**b**) or inflammatory RNA levels (**c**) in 12 h LPS-stimulated RAW 264.7 macrophages. Graphs on right of the IF images representing the percentage of cells with indicated nucleoli numbers per cell. *n*, total cells counted. DAPI, blue. Scale bars, 5 μm. **d**, Graphs representing influence of FL-NCL-GFP or NCLΔAS-GFP overexpression on inflammatory RNA levels in *Ncl*-depleted RAW 264.7 cells after 12 h of LPS stimulation. Overexpression of NCL constructs was assessed by immunoblot with anti-GFP or anti-NCL antibodies. *P* values are determined by unpaired two-tailed *t*-test. ***P*<0.01 (Student's *t*-test). Data are representative of three independent experiments and are presented as means ± s.d. in **b-d**. Source data are provided as a Source Data file.

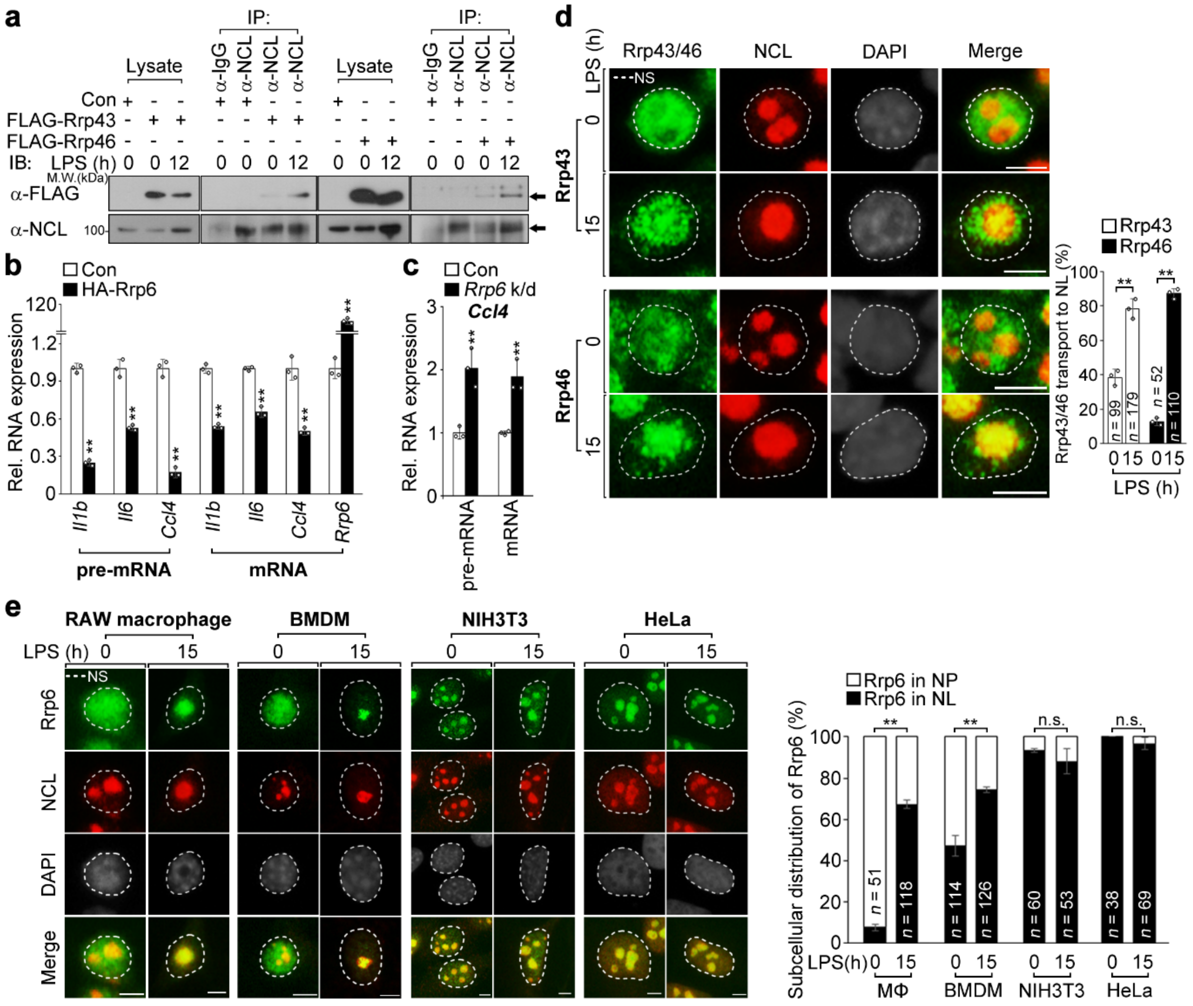

Supplementary Figure 8 | Both binding activity of NCL and nucleolar targeting properties of Rrp43 and Rrp46 are only occurred by LPS stimulation. a, Interaction of NCL with Rrp43 or Rrp46 occurred only in LPS-stimulated RAW 264.7 macrophages expressing FLAG-tagged Rrp43 or Rrp46. Arrows represent the corresponding proteins detected by indicated antibodies. **b**, **c**, Effects of HA-tagged Rrp6 overexpression (**b**) or *Rrp6* depletion (**c**) on inflammatory RNA expression levels in 12 h LPS-stimulated RAW 264.7 macrophages. **d**, Imaging showing nucleolar enrichment of Rrp43 or Rrp46 in LPS-stimulated FLAG-Rrp43/46-expressing RAW 264.7 macrophages. FLAG-Rrp43/46 and NCL were detected by anti-FLAG and anti-NCL antibodies, respectively. The white dashed lines delineate the borders of nucleus (NS) based on DAPI. Graphs on right of the images representing quantification of cells appearing nucleolar enrichment of Rrp proteins. *n*, total cells counted. Scale bars, 5 μm. **e**, Nucleolar enrichment of Rrp6 and NCL in LPS-stimulated RAW 264.7 macrophages (MΦ) (80 ng ml^{-1}), BMDMs (80 ng ml^{-1}), NIH3T3 (80 ng ml^{-1}), and HeLa (1 μg ml^{-1}). Subcellular localization of Rrp6 or NCL was observed by anti-Rrp6 or anti-NCL antibody, respectively. Scale bars, 5 μm. Quantification of Rrp6 nucleolar enrichment for images is shown in right graphs. For details of quantitative measurement of colocalization, see Methods. *P* values are determined by unpaired two-tailed *t*-test. $^{**}P$<0.01 (Student's *t*-test). n.s., not significant. Data are representative of three independent experiments and are presented as means ± s.d. in **b-e**. Source data are provided as a Source Data file.

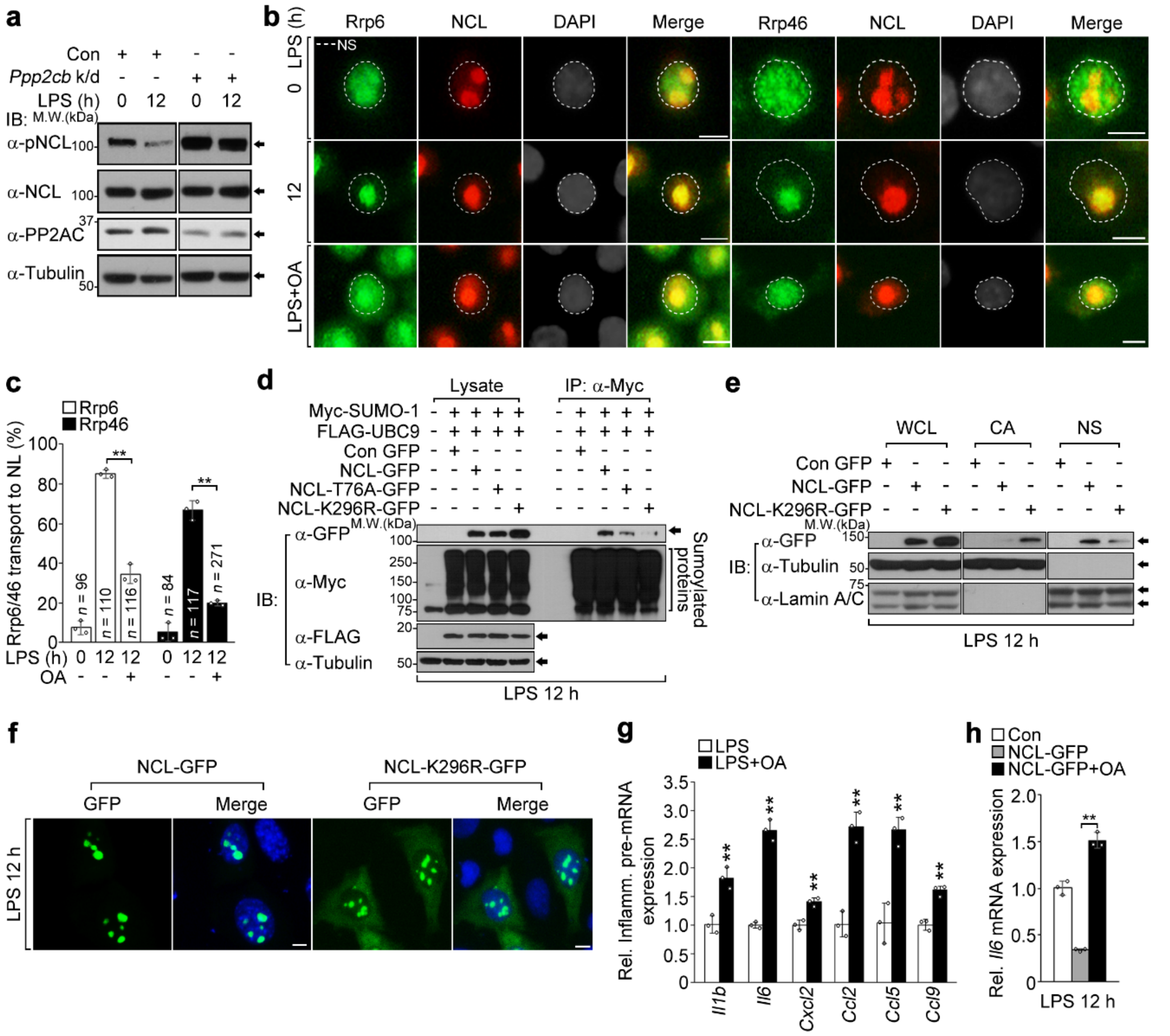

Supplementary Figure 9 | Dephosphorylated and sumoylated NCL promotes nucleolar targeting of Rrp6 and inflammatory RNA instability. a, Immunoblots showing restoration of NCL phosphorylation levels in *Ppp2cb*-depleted RAW264.7 cells under LPS stimulation. **b**, Imaging showing effects of OA on nucleolar translocation of Rrp6 or Rrp46 in macrophages expressing mock or FLAG-Rrp46 under 12 h LPS stimulation with or without OA. Subcellular localization of Rrp6 or Rrp46 was detected by anti-Rrp6 or anti-FLAG antibody, respectively. The white dashed lines delineate the borders of nucleus (NS) based on DAPI. Scale bars, 5 µm. **c**, Graphs representing the percentage of cells exhibiting nucleolar enrichment of Rrp6 or Rrp46 in 20 nM OA-treated macrophages under 12 h LPS stimulation. *n*, total cells counted. **d**, Analysis of NCL sumoylation in Myc-SUMO-1- and FLAG-UBC9-expressing MEFs that transiently expressed wild-type NCL-, NCL-T76A- or NCL-K296R-GFP in the presence of LPS. **e**, **f**, Influence of GFP-tagged wild-type or mutant NCL (NCL-GFP or NCL-K296R-GFP) on its subcellular localization in wild-type NCL- or NCL-K296R-GFP-expressing MEFs under 12 h LPS stimulation. WCL, whole cell lysates; CA, cytoplasmic fractions; NS, nuclear fractions. DAPI, blue. Scale bars, 5 µm. **g**, **h**, Graphs showing effects of OA on inflammatory pre-mRNAs in 12 h LPS-stimulated RAW 264.7 macrophages (**g**) or on *Il6* levels in NCL-GFP overexpressing RAW 264.7 cells under 12 h LPS stimulation (**h**). Arrows represent the corresponding proteins detected by indicated antibodies. *P* values are determined by unpaired two-tailed *t*-test. ***P*<0.01 (Student's *t*-test). Data are representative of three independent experiments and are presented as mean ± s.d. in **c** and **g-h**. Source data are provided as a Source Data file.

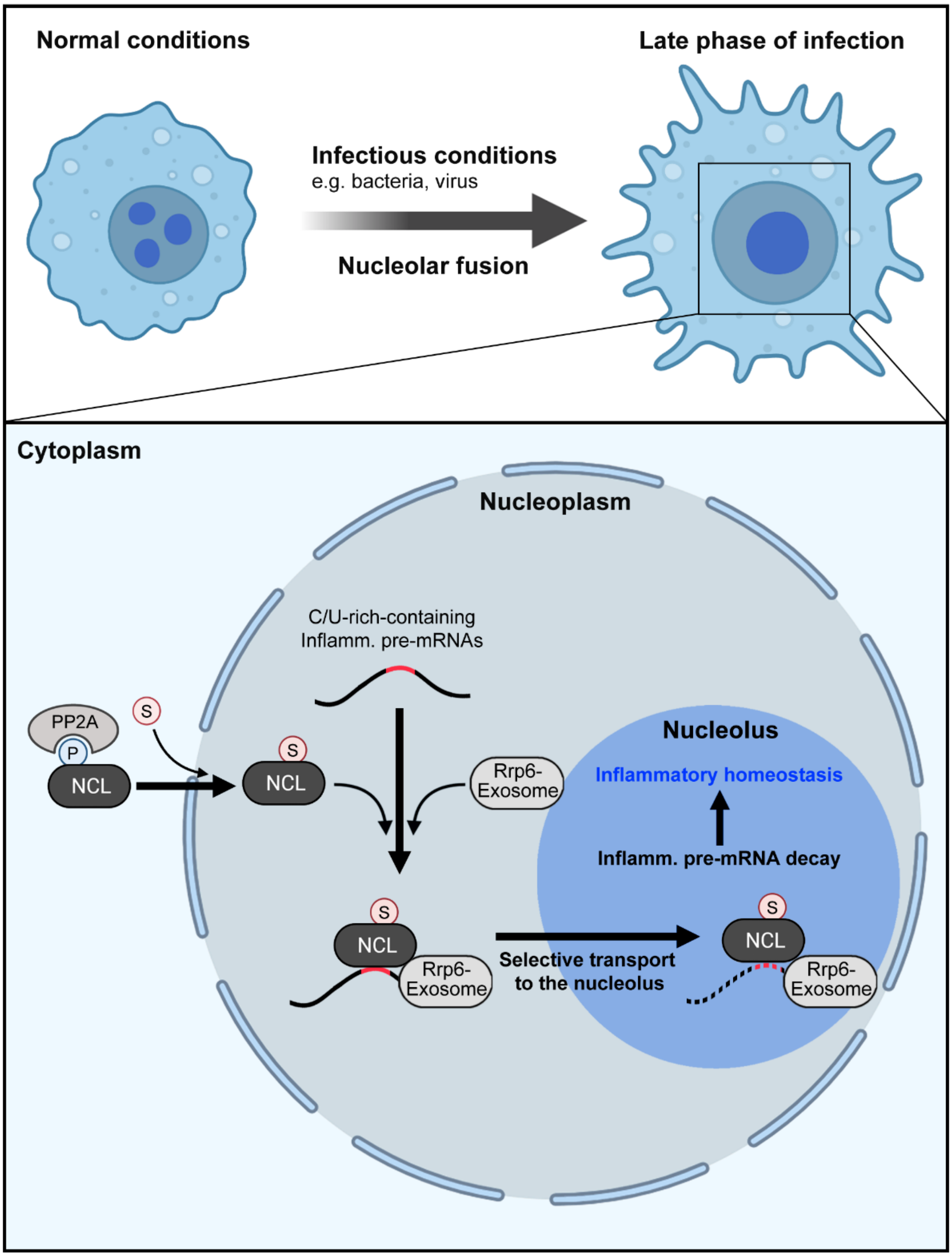

Supplementary Figure 10 | A proposed model depicting the nucleolus as an essential organelle for the instability of inflammatory pre-mRNAs. The nucleolus plays a critical role in modulating the instability of inflammatory pre-mRNAs. At later times of infection, inflammatory pre-mRNAs are preferentially targeted and accumulate in the nucleolus and their expression is tightly controlled by NCL-Rrp6-exosome-dependent pre-mRNA decay occurred in the nucleolus. NCL is dephosphorylated by PP2A and subsequently sumoylated, which is crucial for not only binding Rrp6-exosomes as well as C/U-rich-containing inflammatory pre-mRNAs, but transporting them to the nucleolus. This PTM dynamics eventually leads to degradation of NCL-bound inflammatory pre-mRNAs in the nucleolus, thereby maintaining immune homeostasis. The proposed model was created with BioRender.com.
