# Supplementary material for: The nucleolus is the site for inflammatory RNA decay during infection
Source: Nat Commun. 2022 Sep 3;13:5203. doi: 10.1038/s41467-022-32856-2 (PMC9440930; doi:10.1038/s41467-022-32856-2)
Supplement: Supplementary file 3 — Description of Additional Supplementary Files [file 41467_2022_32856_MOESM3_ESM.pdf]

Description of Additional Supplementary Files

File Name: **Supplementary Data 1**
Description: Inflammatory and non-inflammatory genes in the cytoplasm (CA), nucleoplasm (NP), and nucleoli (NL) for 0, 2, 6, 12, and 15 h of LPS stimulation (Sheet #1-6). List of NCL targets and non-targets analyzed by PAR-CLIP (Sheet #7-8).

File Name: **Supplementary Data 2**
Description: NCL-binding sites and sequences analyzed by PAR-CLIP.

File Name: **Supplementary Data 3**
Description: Reagents, antibodies, primer sequences, RNA-FISH probes, northern blot probes, EMSA probes, and siRNA sequences used in this study.

File Name: **Supplementary Movie 1**
Description: Time-lapse monitoring of green fluorescence protein (GFP)-tagged fibrillarin (FBL).
