# Supplementary material for: The nucleolus is the site for inflammatory RNA decay during infection
Source: Nat Commun. 2022 Sep 3;13:5203. doi: 10.1038/s41467-022-32856-2 (PMC9440930; doi:10.1038/s41467-022-32856-2)
Supplement: Supplementary file 8 — Reporting Summary [file 41467_2022_32856_MOESM8_ESM.pdf]

nature portfolio

Corresponding author(s): Je-Hyun Yoon, Sungwook Lee, Boyoun Park

Last updated by author(s): Jul 14, 2022

Reporting Summary

Nature Portfolio wishes to improve the reproducibility of the work that we publish. This form provides structure for consistency and transparency in reporting. For further information on Nature Portfolio policies, see our Editorial Policies and the Editorial Policy Checklist.

Statistics

For all statistical analyses, confirm that the following items are present in the figure legend, table legend, main text, or Methods section.

n/a	Confirmed	
☐	☒	The exact sample size (n) for each experimental group/condition, given as a discrete number and unit of measurement
☐	☒	A statement on whether measurements were taken from distinct samples or whether the same sample was measured repeatedly
☐	☒	The statistical test(s) used AND whether they are one- or two-sided<br>*Only common tests should be described solely by name; describe more complex techniques in the Methods section.*
☒	☐	A description of all covariates tested
☐	☒	A description of any assumptions or corrections, such as tests of normality and adjustment for multiple comparisons
☐	☒	A full description of the statistical parameters including central tendency (e.g. means) or other basic estimates (e.g. regression coefficient) AND variation (e.g. standard deviation) or associated estimates of uncertainty (e.g. confidence intervals)
☐	☒	For null hypothesis testing, the test statistic (e.g. F, t, r) with confidence intervals, effect sizes, degrees of freedom and P value noted<br>*Give P values as exact values whenever suitable.*
☒	☐	For Bayesian analysis, information on the choice of priors and Markov chain Monte Carlo settings
☒	☐	For hierarchical and complex designs, identification of the appropriate level for tests and full reporting of outcomes
☒	☐	Estimates of effect sizes (e.g. Cohen's d, Pearson's r), indicating how they were calculated

Our web collection on statistics for biologists contains articles on many of the points above.

Software and code

Policy information about availability of computer code

Data collection	No software was used for data collection
Data analysis	Zen blue, MS Excel 2016, photoshop CS5, ExpressionSuite Software, GraphPad Prism 8.0 , Multiple experiment viewer (v.4.9.0), Tophat2 (v.2.1.1), Bowtie2 (v.2.2.9), Integrative Genomics Viewer (IGV) v.2.3.93, PARpipe, PARalyzer (v.1.5), MEME-ChIP (v.5.0.4), MASCOT (v.2.2.04).

For manuscripts utilizing custom algorithms or software that are central to the research but not yet described in published literature, software must be made available to editors and reviewers. We strongly encourage code deposition in a community repository (e.g. GitHub). See the Nature Portfolio guidelines for submitting code & software for further information.

Data

Policy information about availability of data

All manuscripts must include a data availability statement. This statement should provide the following information, where applicable:

- Accession codes, unique identifiers, or web links for publicly available datasets
- A description of any restrictions on data availability
- For clinical datasets or third party data, please ensure that the statement adheres to our policy

All data generated or analyzed during this study are included in this published article (and its supplementary information files).

Field-specific reporting

Please select the one below that is the best fit for your research. If you are not sure, read the appropriate sections before making your selection.

☒ Life sciences ☐ Behavioural & social sciences ☐ Ecological, evolutionary & environmental sciences

For a reference copy of the document with all sections, see nature.com/documents/nr-reporting-summary-flat.pdf

Life sciences study design

All studies must disclose on these points even when the disclosure is negative.

Sample size	No statistical method was used to predetermine sample sizes. Sample sizes are based on the number of replicates necessary to achieve statistical significance derived from previous studies and our experiences.
Data exclusions	No data were excluded from the analysis.
Replication	Results were consistently replicated across experiments as indicated in figure legends
Randomization	Mices were assigned randomly to experimental and control groups. Other experiments were not relevant as there were not grouping used in this study.
Blinding	Investigators were not blinded to allocation during experiments and outcome assessment.

Reporting for specific materials, systems and methods

We require information from authors about some types of materials, experimental systems and methods used in many studies. Here, indicate whether each material, system or method listed is relevant to your study. If you are not sure if a list item applies to your research, read the appropriate section before selecting a response.

Materials & experimental systems

n/a	Involved in the study	
☐	☒	Antibodies
☐	☒	Eukaryotic cell lines
☒	☐	Palaeontology and archaeology
☐	☒	Animals and other organisms
☒	☐	Human research participants
☒	☐	Clinical data
☒	☐	Dual use research of concern

Methods

n/a	Involved in the study	
☒	☐	ChIP-seq
☒	☐	Flow cytometry
☒	☐	MRI-based neuroimaging

Antibodies

Antibodies used	All antibodies used in the study have been listed in supplementay table 3 (Manufacturer, catalog number).<br>Anti-nucleolin (NCL) Santa Cruz Biotechnology #sc-13057 IP (0.6 µg), WB (1:2000), IFA (1:200)<br>Anti-nucleolin (NCL) Abcam #ab22758 IP (0.4 µg), WB (1:2000), IFA (1:200)<br>Anti-phospho-NCL (Thr76) Abcam #ab168363 WB (1:1000)<br>Anti-Rrp6 Santa Cruz Biotechnology #sc-374595 WB (1:1000), IFA (1:200)<br>Anti-Fibrillarin (FBL) Abcam #ab5821 RNA-FISH (1:200)<br>Anti-Fibrillarin (FBL) Novus biologicals #NB300-269 WB (1:1000), IFA (1:200)<br>Anti-β-actin Santa Cruz Biotechnology #sc-8432 WB (1:2000)<br>Anti-Tubulin Applied Biological Materials #G094 WB (1:3000)<br>Anti-PP2AC Merck Millipore #05-421 WB (1:2000)<br>Anti-Lamin A/C Cell Signaling Technology #2032 WB (1:1000)<br>Anti-H2AX Abcam #ab11175 WB (1:2000)<br>Anti-TDP-43 Bethyl #A303-223A WB (1:2000)<br>Anti-UBF Santa Cruz Biotechnology #sc-13125 IFA (1:200)<br>Anti-RNA Pol II Phosphorylation at serine 2 (anti-pS2) Abcam #ab5095 RNA-FISH (1:200)<br>Anti-FLAG Applied Biological Materials #G191 WB (1:1000), IFA (1:200)<br>Anti-Myc Cell Signaling Technology #9B11 WB (1:1000)<br>Anti-GFP Roche #11814460001 IP (0.6 µg)<br>Anti-GFP Santa Cruz Biotechnology #sc-9996 WB (1:2000)
Validation	All commercial antibodies were tested by the supplier.<br>Rabbit anti-Nucleolin – Santa Cruz Biotechnology – validated in 294 publications referenced<br>Rabbit anti-Nucleolin – Abcam – validated in 157 publications referenced

Rabbit anti-phospho-Nucleolin (Thr76) – Abcam – validated in 2 publications referenced
Mouse anti-Rrp6 – Santa Cruz Biotechnology – validated in 8 publications referenced
Rabbit anti-Fibrillarin (FBL) – Abcam – validated in 176 publications referenced
Mouse anti-Fibrillarin (FBL) – Novus biologicals – validated in 16 publications referenced
Mouse anti-β-actin – Santa Cruz Biotechnology – validated in 1,983 publications referenced
Mouse anti-Tubulin – Applied Biological Materials – validated in 21 publications referenced
Mouse anti-PP2AC – Merck Millipore – validated in 157 publications referenced
Rabbit anti-Lamin A/C – Cell Signaling Technology – validated in 270 publications referenced
Rabbit anti-H2AX – Abcam – validated in 151 publications referenced
Rabbit anti-TDP-43 – Bethyl – validated in 8 publications referenced
Mouse anti-UBF – Santa Cruz Biotechnology – validated in 154 publications referenced
Rabbit anti-RNA Pol II Phosphorylation at serine 2 (anti-pS2) – Abcam – validated in 478 publications referenced
Mouse anti-FLAG – Applied Biological Materials – validated in 17 publications referenced
Mouse anti-Myc – Cell Signaling Technology – validated in 1,472 publications referenced
Mouse anti-GFP – Roche – validated in 1,708 publications referenced
Mouse anti-GFP – Santa Cruz Biotechnology – validated in 2,453 publications referenced

Eukaryotic cell lines

Policy information about cell lines

Cell line source(s)	RAW 264.7 (TIB-71, ATCC), MEFs (CRL-2907, ATCC), HEK 293T (CRL-11268, ATCC), NIH3T3 (CRL-1658, ATCC), HeLa (CCL-2, ATCC)
Authentication	These are authenticated and immortalized classic cell line, available at ATCC.
Mycoplasma contamination	Cell lines tested negative for mycoplasma.
Commonly misidentified lines (See ICLAC register)	No commonly misidentified cell lines were used in the study.

Animals and other organisms

Policy information about studies involving animals; ARRIVE guidelines recommended for reporting animal research

Laboratory animals	C57BL/6N mice (4–8 weeks, OrientBio) were maintained in the specific pathogen-free facility according to Korean Food and Drug Administration guidelines.
Wild animals	No wild animals were used in the study.
Field-collected samples	No field collected samples were used in the study.
Ethics oversight	All animal experiments were reviewed and approved by the Institutional Animal Care and Use Committee of the Yonsei University.

Note that full information on the approval of the study protocol must also be provided in the manuscript.
