# Supplementary material for: The nucleolus is the site for inflammatory RNA decay during infection
Source: Nat Commun. 2022 Sep 3;13:5203. doi: 10.1038/s41467-022-32856-2 (PMC9440930; doi:10.1038/s41467-022-32856-2)

Uncropped images of agarose gels and immunoblots

Fig. 4b

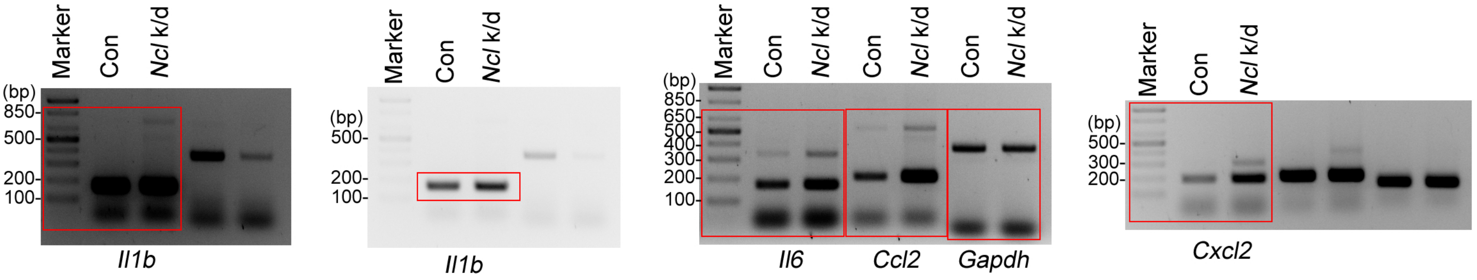

Fig. 5d

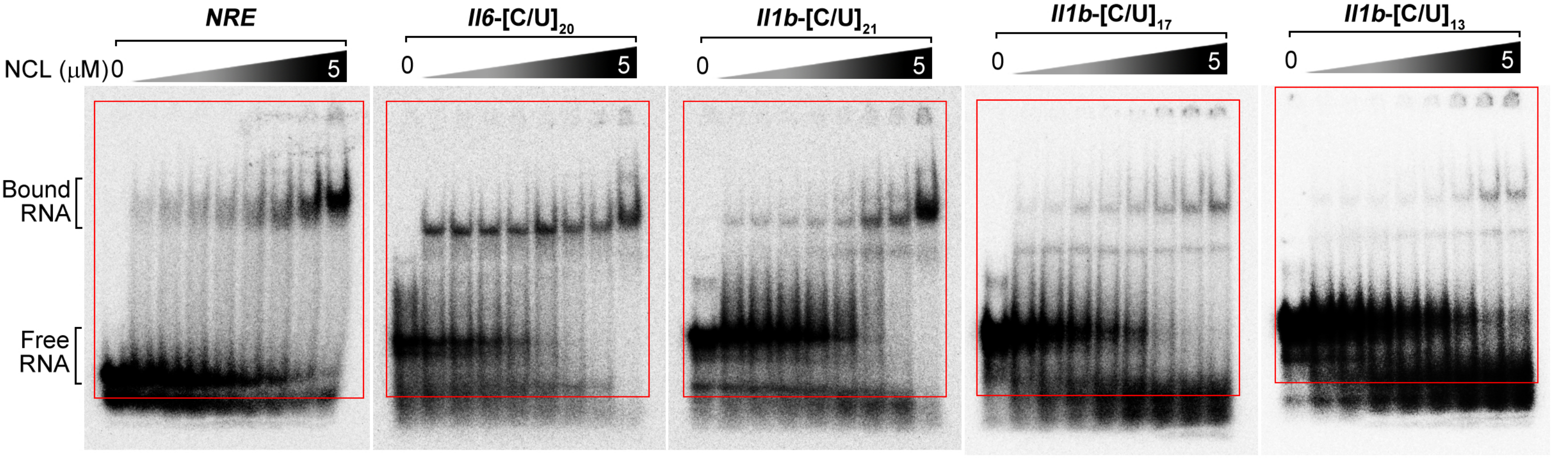

Fig. 6a

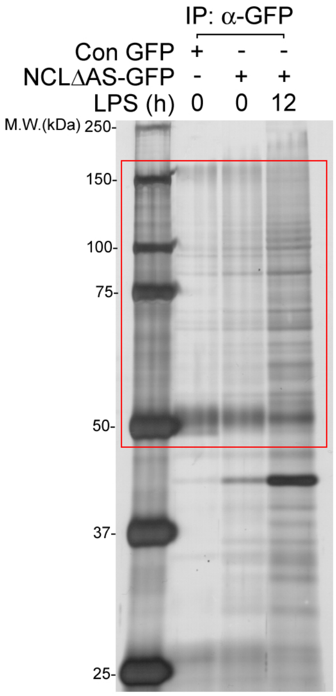

Fig. 6b

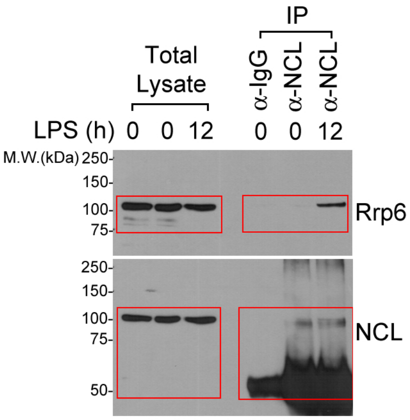

Fig. 6e

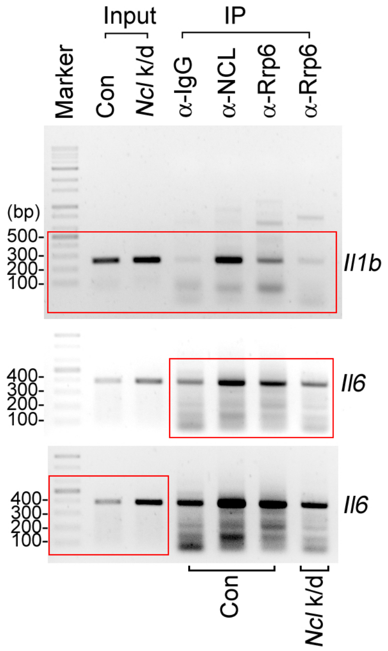

Uncropped images of agarose gels and immunoblots (Continuation)

Fig. 7b

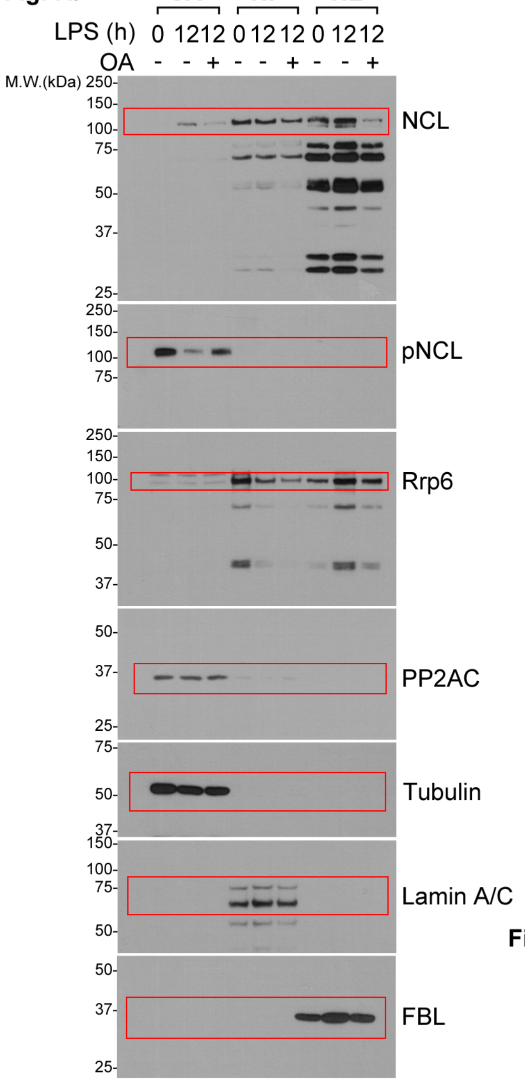

Fig. 7c

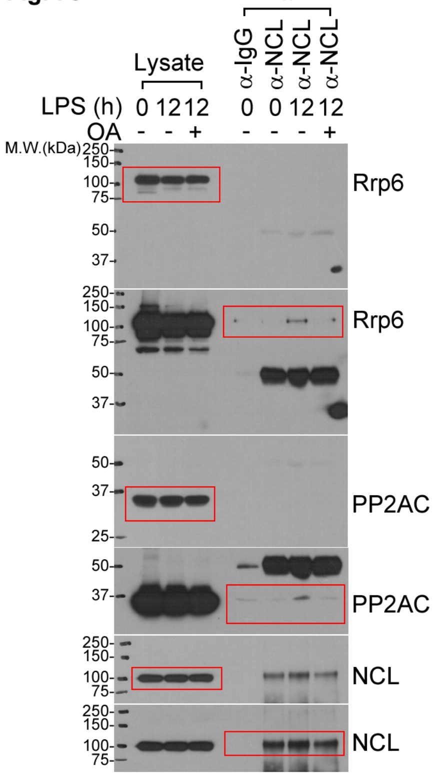

Fig. 7d

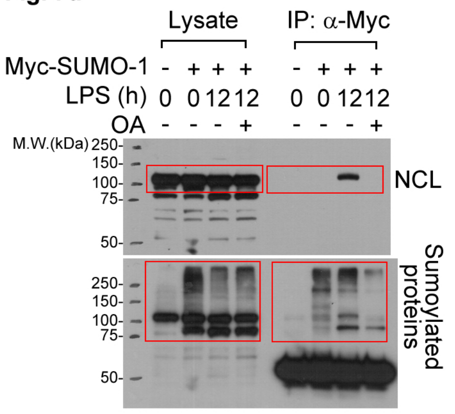

Fig. 7e

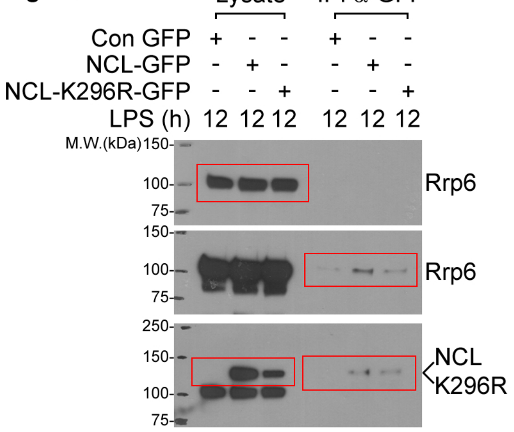

Fig. 7g

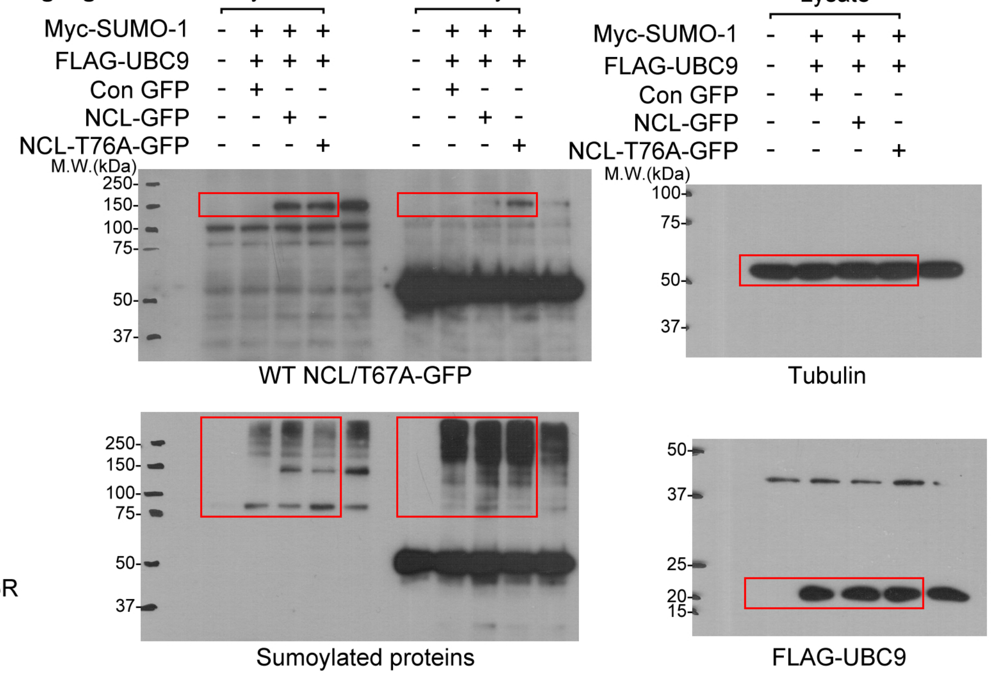

Uncropped images of agarose gels and immunoblots (Continuation)

Supplementary Fig. 2b

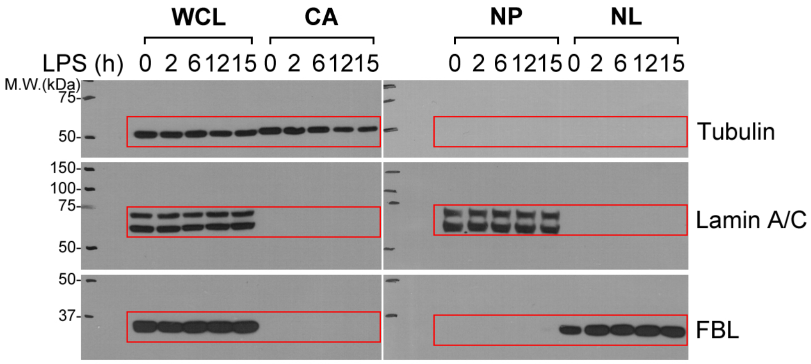

Supplementary Fig. 3b

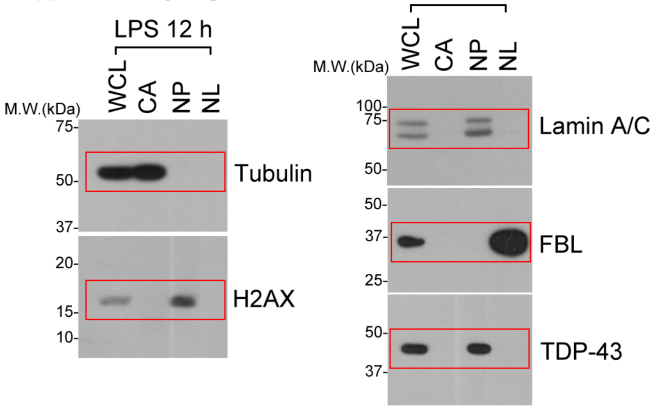

Supplementary Fig. 4c

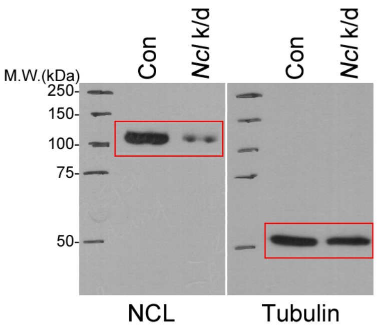

Supplementary Fig. 4d

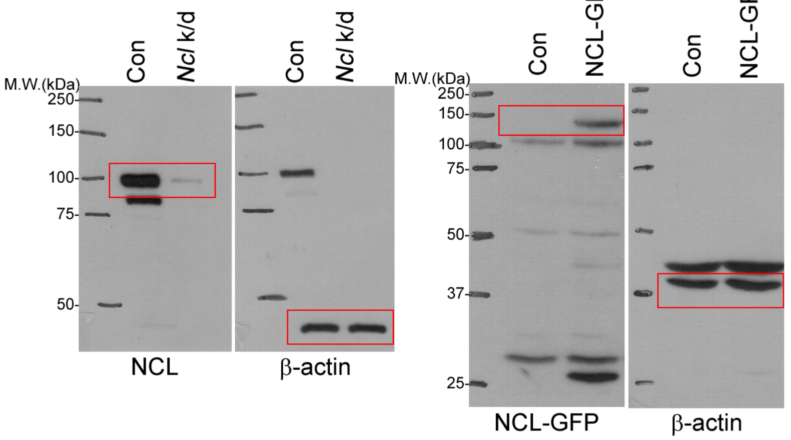

Supplementary Fig. 4e

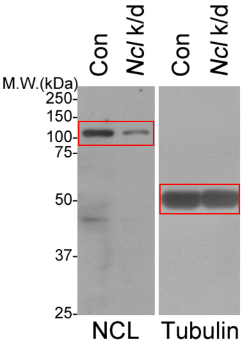

Supplementary Fig. 5a

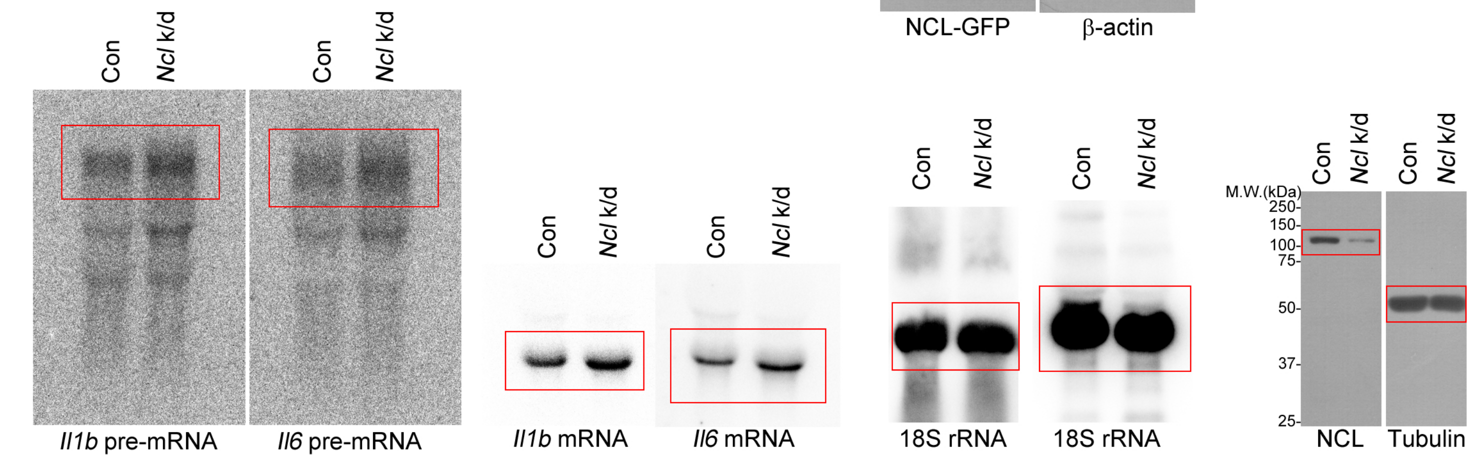

Supplementary Fig. 5c

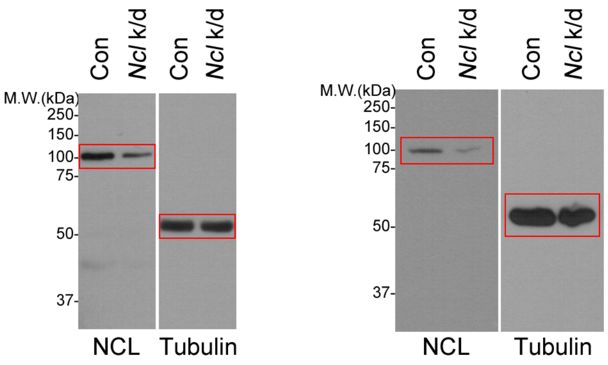

Supplementary Fig. 6a

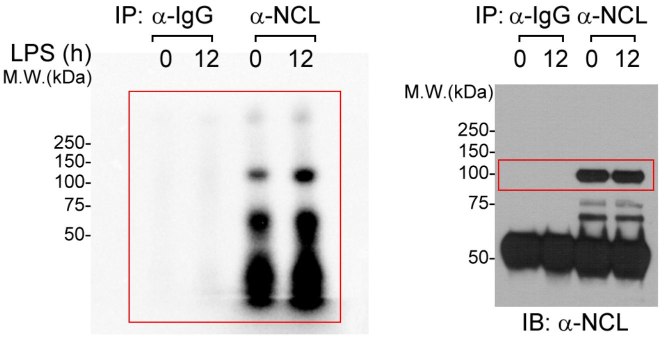

Uncropped images of agarose gels and immunoblots (Continuation)

Supplementary Fig. 7d

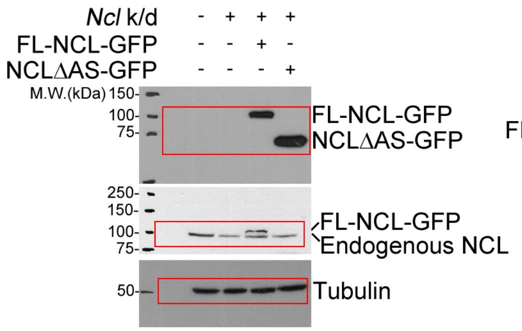

Supplementary Fig. 8a

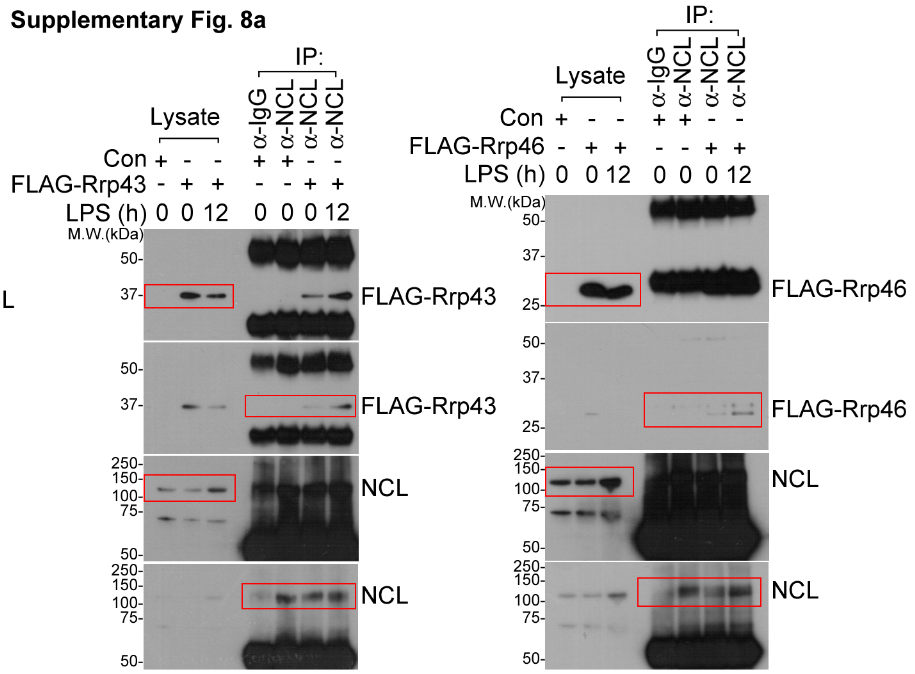

Supplementary Fig. 9a

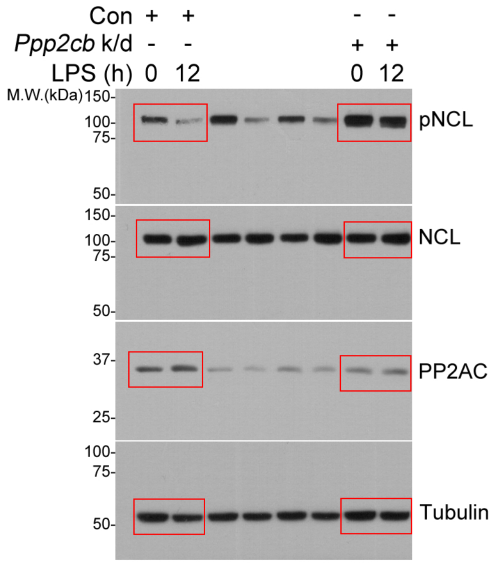

Supplementary Fig. 9e

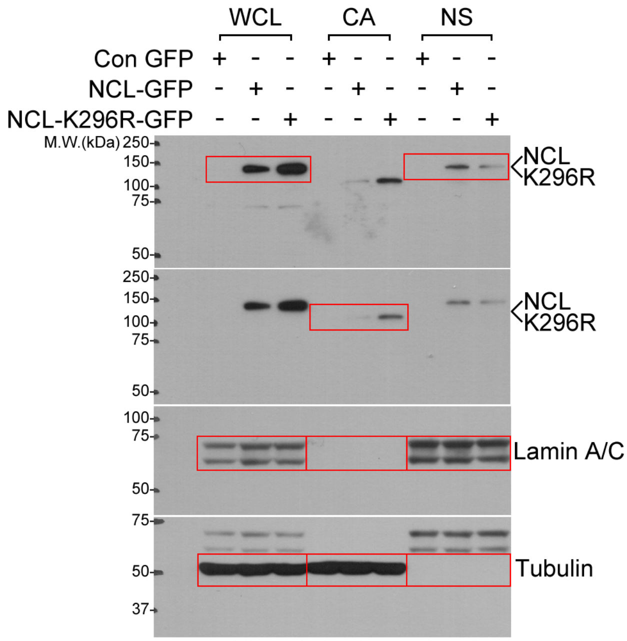

Supplementary Fig. 9d

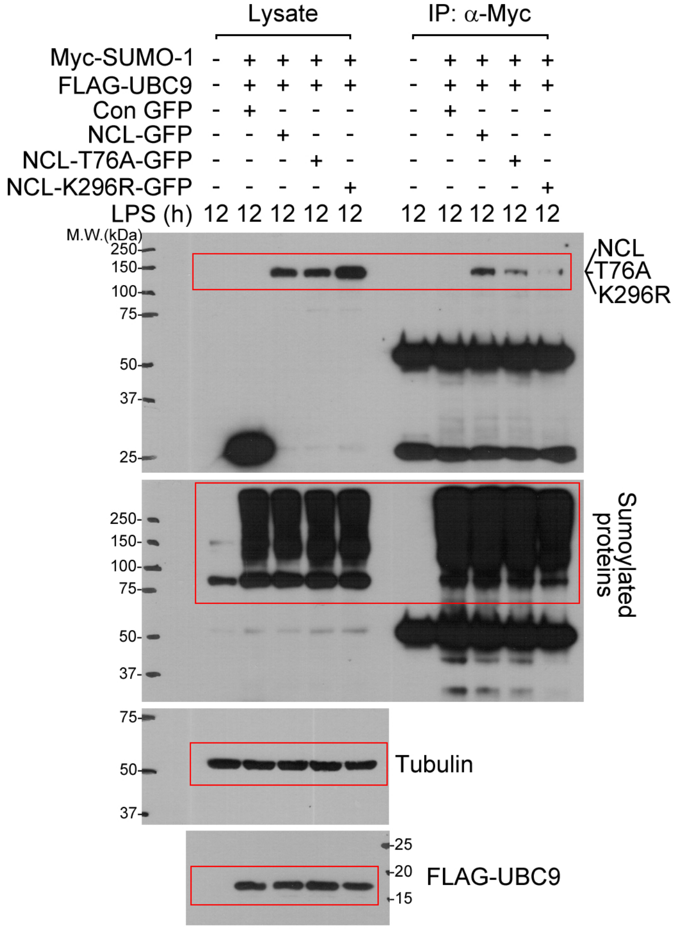

Supplement: Supplementary file 9 — Source Data [file 41467_2022_32856_MOESM9_ESM.zip › Source data for uncropped gels.pdf]
